# Size-dependent catalytic and melting properties of platinum-palladium nanoparticles

**DOI:** 10.1186/1556-276X-6-396

**Published:** 2011-05-26

**Authors:** Grégory Guisbiers, Gulmira Abudukelimu, Djamila Hourlier

**Affiliations:** 1 Institute of Mechanics, Materials and Civil Engineering, Catholic University of Louvain, 2 Place Sainte Barbe, 1348 Louvain-La-Neuve, Belgium; 2 Yili Normal University, 298 Jie Fang Lu Street, Yi Ning Shi, Xinjiang, China; 3 Institute of Electronics, Microelectronics and Nanotechnology, Scientific City, Avenue Henri Poincaré BP60069, 59652 Villeneuve d'Ascq, France

## Abstract

While nanocatalysis is a very active field, there have been very few studies in the size/shape-dependent catalytic properties of transition metals from a thermodynamical approach. Transition metal nanoparticles are very attractive due their high surface to volume ratio and their high surface energy. In particular, in this paper we focus on the Pt-Pd catalyst which is an important system in catalysis. The melting temperature, melting enthalpy, and catalytic activation energy were found to decrease with size. The face centered cubic crystal structure of platinum and palladium has been considered in the model. The shape stability has been discussed. The phase diagram of different polyhedral shapes has been plotted and the surface segregation has been considered. The model predicts a nanoparticle core rich in Pt surrounded by a layer enriched in Pd. The Pd segregation at the surface strongly modifies the catalytic activation energy compared to the non-segregated nanoparticle. The predictions were compared with the available experimental data in the literature.

**PACS:**

65.80-g; 82.60.Qr; 64.75.Jk

## Introduction

Bimetallic nanoparticles exhibit unusual physicochemical properties different from those of the bulk material or their individual constituents [1,2]. They are very used in catalysis, fuel cells, and hydrogen storage. These unusual properties are determined by their size, shape, and composition. When considering metallic catalysts, platinum is a standard material but this material is most expensive than gold [3]. Therefore, to reduce the amount of platinum and then the cost of the application, one possible way is to use an alloy of platinum with another metal. In the present study, the chosen alloy is the binary Pt-Pd system [4] that we propose to theoretically study from a thermodynamic approach [5,6], as well as its pure components. It has been shown previously [5,6] that thermodynamics may provide useful insights in nanotechnology where the size of the considered nanoparticles is higher than approximately 4 nm. Within this approach, the size and shape effects on the melting temperature, melting enthalpy, phase diagram, and catalytic activation energy of this system are investigated.

As face-centered cubic (fcc) metals, Pt and Pd can exhibit a variety of geometrical shapes. Therefore, to address the shape effect on the materials properties of these metals at the nanoscale [7,8], the following shapes have been considered: sphere, tetrahedron, cube, octahedron, decahedron, dodecahedron, truncated octahedron, cuboctahedron, and icosahedron.

### Size-dependent melting properties of Pt and Pd

At the nanoscale, the melting temperature *T*_*m *_and melting enthalpy Δ*H*_*m*_, for free-standing nanostructures can be expressed as function of their bulk corresponding property, the size of the structure and one shape parameter [9].(1)(2)

where the shape parameter, α_shape_, is defined as *α*_shape _= *AD*(*γ*_*s*_-*γ*_*l*_)/(*V*Δ*H*_*m*, ∞_); *D *being the size of the structure (*i.e. *for a sphere, *D *is the diameter), *A *(meter squared) and *V *(cubic meter) are the surface area and volume of the nanostructure, respectively. Δ*H*_*m,∞ *_is the bulk melting enthalpy (Joule per cubic meter), whereas *γ*_*l *_and *γ*_*s *_are the surface energy in the liquid and solid phases (Joule per square meter), respectively. *γ*_*l *_and *γ*_*s *_are considered size independent. This is justified by the fact that the size effect on the surface energies is less than 4% for sizes higher than 4 nm [10,11]. Indeed, below this size, edges, and corners of the structures begin to play a significant role in the surface energy [12].

**Figure 1:**
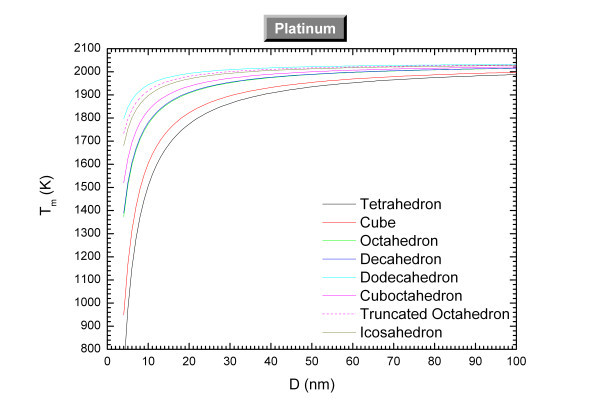
**Size-dependent melting temperature of platinum *versus *the size for different shapes**.

**Figure 2:**
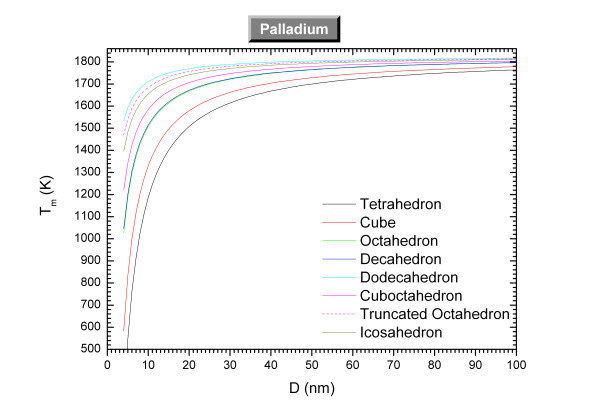
**Size-dependent melting temperature of palladium *versus *the size for different shapes**.

The size-dependent melting temperatures of platinum and palladium are plotted in Figures 1 and 2 respectively. The materials properties of the considered materials are indicated in Table 1. The melting properties for the sphere have been calculated using for the solid surface energy the mean value of experimental data [13]. For the other polyhedra shapes, we have considered the fcc crystal structure of the metals and the respective solid surface energy for each face [14]. Tables 2 and 3 indicate the parameters used for the calculation of the melting properties. Experimentally, the melting of agglomerated Pt nanocrystals (tetrahedrons and cubes) with an average size around approximately 8 nm starts at approximately 900 K [15] in relative good agreement with our theoretical predictions. Molecular dynamics simulations [16] have calculated the size effect on the melting temperature of Pd and found α_sphere _= 0.95 *nm *while our theory predicts 1.68 nm.

**Table 1:** Materials properties of platinum and palladium.

Materials properties	Platinum	Palladium
*T*_*m,*∞ _(K) [40]	2,041.5	1,828

*ΔH*_*m,*∞ _(kJ/mol) [40]	22	17

Δ*H*_sub, ∞ _(kJ/mol) [41]	565	377

*γ*_*l *_(J/m^2^)[40]	1.866	1.470

*γ*_*s *_(J/m^2^) [13]	2.482	2.027

**Table 2:** Solid surface energies for platinum and palladium materials [13].

Faces	Platinum	Palladium
*γ*_*s *_(111) (J/m^2^)	2.299	1.920

*γ*_*s *_(100) (J/m^2^)	2.734	2.326

*γ*_*s *_(110) (J/m^2^)	2.819	2.225

**Table 3:** Number of (hkl) faces for each shape.

Shape	Number of (111) faces	Number of (100) faces	Number of (110) faces
Tetrahedron	4	0	0

Cube	0	6	0

Octahedron	8	0	0

Decahedron	10	0	0

Dodecahedron	12	0	0

Truncated octahedron	8	6	0

Cuboctahedron	8	6	0

Icosahedron	20	0	0

## Discussion

At the nanoscale, the shape which exhibits the highest melting temperature is the one which minimizes the most the Gibbs' free energy (*G *= *H - TS*); and is then the favored one. From Figures 1 and 2, the four most-stable shapes among the ones considered are the dodecahedron, truncated octahedron, icosahedron, and the cuboctahedron. Experimentally, truncated octahedron and cuboctahedron are observed for platinum nanoparticles [8] whereas icosahedron, decahedron, truncated octahedron and cuboctahedron are observed for palladium nanoparticles [8]. Therefore, our predictions are in relative good agreement with the observations for palladium and platinum except that dodecahedron and icosahedron are not observed for platinum. Other theoretical calculations confirmed that the dodecahedron is a stable shape for palladium [17]. More generally, according to Yacaman *et al. *[8], the most often observed shapes at the nanoscale are the cuboctahedron, icosahedron, and the decahedron.

Furthermore, care has to be taken when we compare theoretical results with experimental ones due those materials properties depend on the synthesis process [18,19]. And then predicted properties from thermodynamics may differ from the experimentally observed if the synthesis process is not running under thermodynamical equilibrium. Moreover, thermal fluctuations are often observed in nanoparticles [20] meaning that the shape stability is much more complicated than just a minimisation of the *A/V *ratio with faces exhibiting the lowest surface energy.

### Nano-phase diagram of Pt-Pd

According to the Hume-Rothery's rules, platinum and palladium forms an ideal solution [21]. In this case, considering no surface segregation, the liquidus and solidus curves of bulk and nanostructures are calculated from the following equations [22-24]:(3)

where *x*_solidus _(*x*_liquidus_) is the composition in the solid (liquid) phase at a given *T*, respectively.  is the size-dependent melting temperature of the element *i*.  is the size-dependent melting enthalpy of the element *i*.

**Figure 3:**
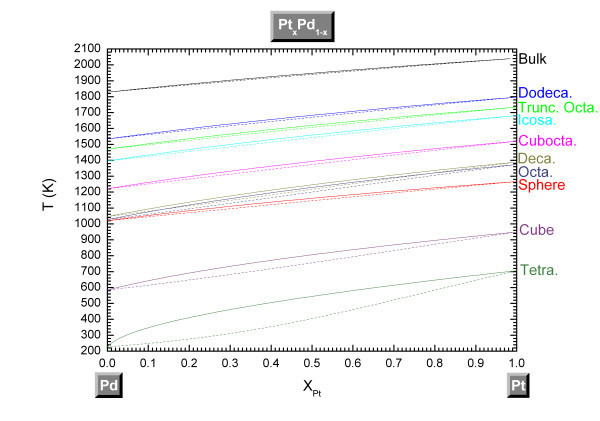
**Phase diagram of the Pt-Pd system for different shapes**. Different shapes at a size equal to 4 nm and at the bulk scale. The solid lines indicate the liquidus curves whereas the dashed lines indicate the solidus ones.

The phase diagram of the Pt-Pd alloy is plotted in Figure 3. We note that the lens shape of the phase diagram is conserved at the nanoscale; however, the lens width increases for the shapes characterized by a small melting enthalpy and melting temperature, *i.e.*, exhibiting a strong shape effect. Moreover, the melting temperature increases with the concentration of Pt in agreement with Ref. [25].

In order to predict nanomaterials properties more accurately, we are considering a possible surface segregation which is known as the surface enrichment of one component of a binary alloy. At the nanoscale, surface segregation leads to a new atomic species repartition between the core and the surface. According to Williams and Nason [26], the surface composition of the liquid and solid phase are given by:(4)

where *z*_1 _is the first nearest neighbor atoms; *z*_1*ν *_is the number of first nearest atoms above the same plane (vertical direction). In the case of face-centered cubic (fcc) crystal structure of Pt and Pd materials, we have *z*_1 _= 12, *z*_1*ν *_= 4 for (100) faces and three for (111) faces. Δ*H*_vap _is the difference between the bulk vaporization enthalpies of the two pure elements, . Δ*H*_sub _is the difference between the bulk sublimation enthalpies of the two pure elements, . Element *A *is chosen to be the one with the highest sublimation and vaporization enthalpies. If the two components are identical, Δ*H*_sub _= 0 and Δ*H*_vap _= 0, there is no segregation and we retrieve Equation 3. *x*_solidus _and *x*_liquidus _are obtained from solving Equation 3. Assuming an ideal solution, only the first surface layer will be different from the core composition.

**Figure 4:**
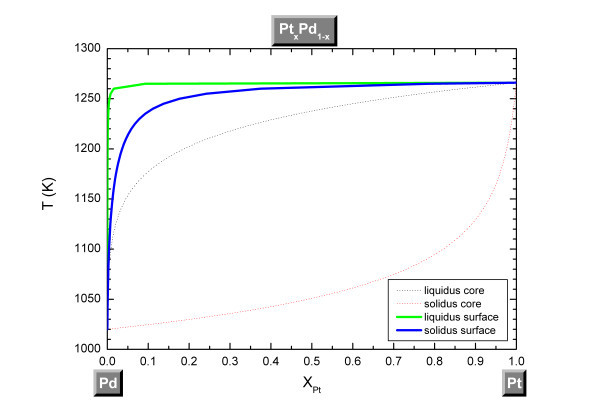
**Phase diagram of the Pt-Pd system considering the surface segregation effect**. Surface segregation effect at a size equal to 4 nm for a spherical nanoparticle.

Considering the surface segregation in the Pt-Pd system, we can see in Figure 4 that the lens shape of the surface liquidus/solidus curves is deformed compared to the core. At a given temperature, the liquidus and solidus curves of the surface are enriched in Pd compared to the core; meaning that the surface is depleted of Pt (the higher bond energy element) which is in agreement with experimental observations[27-29] and other theoretical calculations[29-31]. This is due to the fact that Pd has a lower solid surface energy, a lower cohesive energy compared to Pt and also because diffusion is enhanced at the nanoscale [32].

### Size-dependent catalytic activation energy of Pt-Pd

The catalytic activation energy is the energy quantity that must be overcome in order for a chemical reaction to occur in presence of a catalyst. The low the catalytic activation energy is, the most active the catalyst is. It is thus an important kinetic parameter linked to the chemical activity. Indeed, the catalytic activation energy is a linear function of the work function [33-35]. For pure materials, the catalytic activity depends on the fraction of surface atoms on corners and edges while for binary compounds it depends also on the surface segregation. Recently, it has been showed by Lu and Meng in Ref. [36] that the size-dependent catalytic activation energy, *E*_ca _could be obtained from the following relation:(5)

Therefore, it means that the size-dependent catalytic activation energy decreases with size.

To compare with experimental results, the ratio of the catalytic activation energies between tetrahedral (*D *= 4.8 nm) and spherical (*D *= 4.9 nm) pure platinum nanoparticles has been determined around 0.66 in excellent agreement with the experimental value of 0.62 ± 0.06 announced by Narayanan and El-Sayed [37-39]. Moreover, the ratio of the catalytic activation energies between cubic (*D *= 7.1 nm) and spherical (*D *= 4.9 nm) pure platinum nanoparticles is around 1.01 in relative good agreement with the experimental value of 1.17 ± 0.12 [37-39].

**Figure 5:**
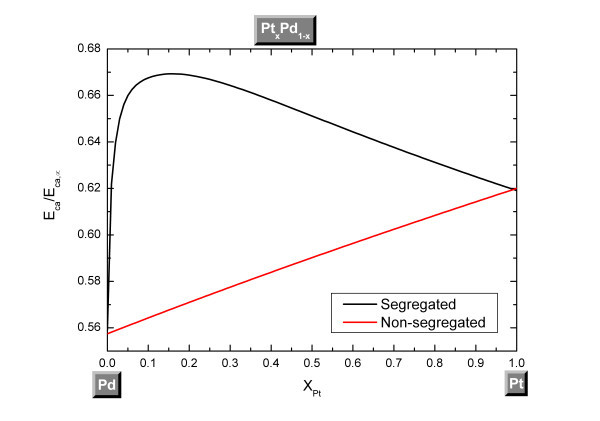
**Composition dependency of the catalytic activation energy for a spherical nanoparticle of Pt-Pd**. Nanoparticle of Pt-Pd with a size equal to 4 nm.

From the size-dependent Pt-Pd phase diagram, the melting temperature of the alloy can be deduced. Equation 6 describes the melting temperature of the bulk Pt-Pd while Equations 7 and 8 describe the nanoscaled melting temperature of a non-segregated and segregated spherical nanoparticle (with a diameter equal to 4 nm), respectively.(6)(7)(8)

where *x *represents the alloy composition. For a spherical Pt-Pd nanoparticle with a diameter equal to 4 nm, by combining Equations 5-8, *E*_ca _seems to evolve quadratically with the composition when the segregation is not considered; which is not the case when the segregation is considered (Figure 5). For the segregated Pt-Pd nanoparticle, a maximum in the catalytic activation energy is reached around 16% of Pt composition.

## Conclusions

In conclusion, it has been shown that thermodynamics can still provide useful insights in nanoscience and more specifically in catalysis. The future development of catalysts and fuel cells is dependent upon our ability to control the size, shape, and surface chemistry of individual nanoparticles. Future theoretical work will have to consider the environment in which the particles are synthesized as well as the preparation method because these parameters can have a great influence on the shape stability and on the catalytic properties.

## Competing interests

The authors declare that they have no competing interests.

## Authors' contributions

GG carried out the calculations on the size and shape effects on the melting temperature, phase diagrams and catalytic activation energy; drafted the manuscript. GA carried out the calculations on the phase diagrams (shape effect) in collaboration with GG. DH carried out the calculations on the phase diagrams (segregation effect) in collaboration with GG. All authors read and approved the final manuscript.
